# Hypothalamic-pituitary-derived melanocortin axis links to myelopoiesis and immunotherapy

**DOI:** 10.1016/j.xinn.2023.100453

**Published:** 2023-05-30

**Authors:** Chao Zhang, Ya-Xiong Tao

**Affiliations:** 1 Fundamental Research Center, Shanghai Yangzhi Rehabilitation Hospital (Shanghai Sunshine Rehabilitation Center), Tongji University, Shanghai 201619, China; 2 Department of Anatomy, Physiology and Pharmacology, College of Veterinary Medicine, Auburn University, Auburn, AL 36849, USA

Melanocortins, including α-melanocyte stimulating hormone (α-MSH), β-MSH, γ-MSH, and adrenocorticotropin (ACTH), are ancient peptides highly conserved in vertebrates and exhibit diverse well-established functions, including dermal pigmentation, adrenal steroidogenesis and cell proliferation, energy homeostasis, and inflammation.^1^ Melanocortins exert their diverse functions through five melanocortin receptors (MCRs), MC1R–MC5R, numbered based on the sequence of their cloning. A PubMed search performed on May 22, 2023, using MC1R, MC2R, MC3R, MC4R, and MC5R as the search term retrieved 1,815, 474, 554, 2270, and 252 entries, suggesting that compared with the other four MCRs, MC5R received less attention.

Originally identified as a major player for lipid production in the sebaceous and preputial glands in 1997, additional sites of expression and physiological functions of MC5R signaling such as stress response, thermoregulation, and lipid metabolism have been elucidated. MC5R also participates in regulating immune homeostasis in the adipocytes, airway epithelial cells, ocular Treg cells, and spleen antigen-presenting cells.^2^

**Figure 1:**
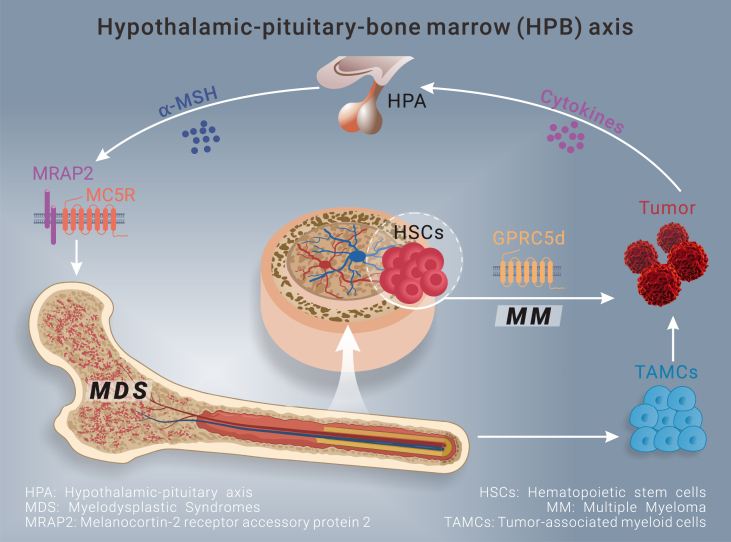
Graphic abstract of hypothalamic-pituitary-bone marrow (HPB) axis Schematic illustration of hypothalamic-pituitary-derived α-MSH in the promotion of bone marrow myelopoiesis, immunotherapy of solid tumors, and potential application for treating MM and MDS in MC5R-dependent manner. HPA: hypothalamic-pituitary-adrenal axis; TAMCs: tumor-associated myeloid cells; HSCs: hematopoietic stem cells; MDS: myelodysplastic syndromes; MM: multiple myeloma; MRAP2: melanocortin-2 receptor accessory protein 2.

On August 4, 2022, Zhou and his colleagues published an intriguing article in *Science*, in which they elucidated a novel role of hypothalamic-pituitary-derived α-MSH and its activation of MC5R signaling in the regulation of myelopoiesis and immunosuppression in cancer-bearing murine models and human patients.^3^ In this study, tumor transplantation results in the activation of neurons in the paraventricular nucleus of the hypothalamus and elevation of serum α-MSH, believed to be derived from the intermediate lobe of the pituitary gland. Proopiomelanocortin (POMC) is processed post-translationally into ACTH in the anterior pituitary and MSHs in the intermediate lobe. Based on the existence of the enzymes involved in the processing, no change of ACTH level suggested that increased α-MSH comes from the intermediate lobe of the pituitary. Whether the POMC neurons in the arcuate nucleus contributed to the elevated α-MSH had not been investigated. Clinically, they observed elevation of serum α-MSH concentration and correlation with circulating myeloid-derived suppressor cells in non-small-cell lung carcinoma and head and neck cancers patients (Figure 1).

Next, they assessed the capability of α-MSH on promoting tumor growth and immune suppression. They showed that knocking down *Pomc* expression increases cytotoxic lymphocyte infiltration in the tumor microenvironment and decreases tumor growth, which is reversed by α-MSH administration. Since α-MSH can activate all four MCRs except MC2R, the expression of only MC5R, but not the other MCRs, in these cells provides a justification for using α-MSH in these experiments. Next, they observed the specific elevation of *Mc5r* expression in the Lineage^−^ bone marrow and abrogation of myeloid progenitor cell expansion in global *Mc5r* knockout or hematopoietic-specific ablated *Mc5r* mice. Administration of MC5R antagonist, Compound 5, dramatically promotes antitumor immunity and enhances anti-PD-1 immunotherapy. It is highly commended that the authors confirmed that Compound 5 is indeed a specific MC5R antagonist, considering recent suggestion that the pharmacology of some peptides might not be accurate.^4^ Whether administration of MC5R-specific agonist shows the opposite effect would be of interest. The very high half maximal inhibitory concentrations in the current study, 0.57 and 2.50 μM for human and mouse MC5Rs, respectively, compared with 10 nM in the original study for human MC5R, are puzzling.

Next, they examined the intracellular signaling of MC5R in the bone marrow. The five MCRs are all G protein-coupled receptors (GPCRs). The classical signaling utilized by GPCRs are heterotrimeric G proteins, producing second messengers such as cyclic AMP, inositol phosphate, and diacyl glycerol, activating protein kinase A and C. JAK/STAT (Janus kinase/signal transducer and activator of transcription) are classically activated by receptors for cytokine and growth factors. There are only a few studies reporting GPCR activation of JAK/STAT pathway. For the MCRs, a previous study reported that Ba/F3 pro-B-lymphocytes express MC5R, and α-MSH stimulates JAK2/STAT1 activation. In the current study, α-MSH activates STAT3, which is blocked by Compound 5, the MC5R antagonist. Whether STAT1 is also activated in the current study is not reported.

As a promising adjuvant GPCR target for treating human cancer, future studies should be focused on the clinical improvement of current immunotherapy by concurrent pharmacological modulation of MC5R signaling. Direct administration of inhibitory antibody, polypeptide antagonist, or small molecule negative allosteric modulator of MC5R might improve cancer therapies through PD1/PDL1, CTLA4, or chimeric antigen receptor T (CAR-T) cells. Recently, numerous novel GPCR partners have been characterized for MC3R/MC4R in the central nervous system.^5^ It is essential to further identify unknown GPCRs that could form hetero-dimers with MC5R in the bone marrow. Co-administration of natural or artificial ligands of these GPCRs could potentially suppress α-MSH-stimulated MC5R signaling in a synergistic manner. In addition, bone-derived lipocalin-2 (LCN2) mediates appetite suppression by directly activating the hypothalamic MC4R signaling in pancreatic cancer cachexia model. Interestingly, Mosialou et al. also observed activation of human MC1R and MC3R with comparable EC_50_ between 1.4 and 1.8 nM by LCN2. The tumor-induced elevation of LCN2 from bone marrow and the potential immunosuppression through activating local MC5R needs to be clarified in subsequent studies. More strikingly, another orphan GPCR (GPRC5D) has been evaluated as a promising target of CAR-T for the treatment of bone marrow-derived multiple myeloma. Follow-up strategies include improvement of the expression of GPRC5D in cancer cells and recognition of surface GPRC5D by CAR-armed T cells. These findings raise intriguing questions on whether α-MSH-induced MC5R signaling in the bone marrow could influence the transcription and surface translocation of GPRC5D proteins. We speculate that the simultaneous inhibition of MC5R through multiple approaches may benefit the GPRC5D CAR-T therapies to treat multiple myeloma.

Serum α-MSH concentration was elevated and correlated with circulating myeloid-derived suppressor cells (MDSCs) in non-small cell lung cancer and head and neck cancer patients. Whether this is a universal phenomenon needs to be observed in many other cancer types. Moreover, MDSCs infiltrated into the tumor microenvironment play a global role to suppress the patient’s immune system and make tumor cells resistant to multiple immunotherapies. Since the activation of MC5R stimulates the proliferation of MDSCs from the bone niche, the human cohorts with natural loss of function of *MC5R* may have decreased incidence of all types of cancers with low plasma level of circulating MDSCs. So far only a few studies of human *MC5R* SNP have been reported, and naturally occurring human *MC5R* mutations recorded in gnomAD database (https://gnomad.broadinstitute.org) were recently illustrated. A large-scale survey on the prevalence epidemiology of all types of malignant tumor in human populations with *MC5R* SNP and the monitoring of the plasma level of circulating MDSCs in these cohorts will ultimately solve this puzzle.

This study is the first report, to our knowledge, to establish a novel physiological link of central neuroendocrine circuit to the peripheral hematopoietic homeostasis in the bone marrow, especially in a MC5R-dependent manner. Therefore, in addition to cancer immunotherapy, clinical development of MC5R as a pharmacological target for the treatment of myelopoiesis-associated disorders deserves extra attention. Myelodysplastic syndrome (MDS) is characterized as a myeloid-derived blood disorder with abnormal development of blood cells within the bone marrow. Immunomodulatory drugs such as thalidomide and lenalidomide or epigenetic therapies including azacitidine and decitabine are currently utilized for the remission of MDS symptoms. If MC5R signaling is indispensable for normal myelopoiesis, it will be worthwhile to examine whether the epigenetic drugs suppress MDS by inhibiting MC5R transcription. An investigator-initiated trial study with MC5R-specific silent hematopoietic stem cell transplantation strategy, achieved by gene editing using CRISPR-Cas9 platform, will be a potential clinical advance for the ultimate cure for MDS patients.

Moreover, an indispensable endogenous physiological chaperone for MCRs has been well defined from previous studies. Melanocortin-2 receptor accessory protein 2 (MRAP2), an essential pharmacological partner for all five melanocortin receptors, is expressed in the myelopoietic cells and hematopoietic tissue of bone marrow (https://www.proteinatlas.org/). Previous evidence demonstrated that human MRAP2 proteins can suppress the plasma membrane translocation and agonist-stimulated cAMP generation of MC5R. As an inhibitory factor, presence of MRAP2 proteins on the cell surface could suppress the myelopoiesis and proliferation of MDSCs caused by MC5R activation. Epidemiological investigation of the incidence of MDS and malignant tumors in human cohorts carrying *MRAP2* loss-of-function SNPs will be inevitable accordingly.

To date, over 150 GPCRs have been explored as drug targets for multiple human diseases, with drugs approved in the United States or the European Union. Pharmacological activation or inhibition of MCRs could potentially treat various pathological disorders including cancer. As a potent agonist for four MCRs (except MC2R), melanotan-II (MT-II) modified ligand-drug conjugates were developed to treat melanoma through the overexpressed MC1R. The FDA also approved bremelanotide (brand name: Vyleesi, 2019) and setmelanotide (brand name: Imcivree, 2020) to treat MC4R-associated hypoactive sexual desire disorder and severe genetic obesity syndrome, respectively. Overall, this finding revealed a novel hypothalamic-pituitary-bone marrow axis as a neuroendocrine pathway that contributed to cancer-induced myelopoiesis and immunosuppression and suggested MC5R as a potential therapeutic target for the treatment of cancers resistant to immune checkpoint therapies in the future.
